# Predicting the Prognosis of Patients in the Coronary Care Unit: A Novel Multi-Category Machine Learning Model Using XGBoost

**DOI:** 10.3389/fcvm.2022.764629

**Published:** 2022-05-12

**Authors:** Xingchen Wang, Tianqi Zhu, Minghong Xia, Yu Liu, Yao Wang, Xizhi Wang, Lenan Zhuang, Danfeng Zhong, Jun Zhu, Hong He, Shaoxiang Weng, Junhui Zhu, Dongwu Lai

**Affiliations:** ^1^Key Laboratory of Cardiovascular Intervention and Regenerative Medicine of Zhejiang Province, Department of Cardiology, Sir Run Run Shaw Hospital, Zhejiang University School of Medicine, Hangzhou, China; ^2^College of Biomedical Engineering and Instrument Science, Zhejiang University, Hangzhou, China

**Keywords:** MIMIC-III, coronary care unit (CCU), machine learning, multi-category, prognosis, XGBoost

## Abstract

**Background:**

Early prediction and classification of prognosis is essential for patients in the coronary care unit (CCU). We applied a machine learning (ML) model using the eXtreme Gradient Boosting (XGBoost) algorithm to prognosticate CCU patients and compared XGBoost with traditional classification models.

**Methods:**

CCU patients' data were extracted from the MIMIC-III v1.4 clinical database, and divided into four groups based on the time to death: <30 days, 30 days−1 year, 1–5 years, and ≥5 years. Four classification models, including XGBoost, naïve Bayes (NB), logistic regression (LR), and support vector machine (SVM) were constructed using the Python software. These four models were tested and compared for accuracy, F1 score, Matthews correlation coefficient (MCC), and area under the curve (AUC) of the receiver operating characteristic curves. Subsequently, Local Interpretable Model-Agnostic Explanations method was performed to improve XGBoost model interpretability. We also constructed sub-models of each model based on the different categories of death time and compared the differences by decision curve analysis. The optimal model was further analyzed using a clinical impact curve. At last, feature ablation curves of the XGBoost model were conducted to obtain the simplified model.

**Results:**

Overall, 5360 CCU patients were included. Compared to NB, LR, and SVM, the XGBoost model showed better accuracy (0.663, 0.605, 0.632, and 0.622), micro-AUCs (0.873, 0.811, 0.841, and 0.818), and MCC (0.337, 0.317, 0.250, and 0.182). In subgroup analysis, the XGBoost model had a better predictive performance in acute myocardial infarction subgroup. The decision curve and clinical impact curve analyses verified the clinical utility of the XGBoost model for different categories of patients. Finally, we obtained a simplified model with thirty features.

**Conclusions:**

For CCU physicians, the ML technique by XGBoost is a potential predictive tool in patients with different conditions, and it may contribute to improvements in prognosis.

## Introduction

Cardiovascular disease (CVD), the leading cause of global mortality and disability, causes ~18.6 million deaths annually. China has the highest mortality worldwide (1). Coronary care units (CCU), which concentrate on the treatment of patients with critical cardiovascular diseases, reduce mortality and prolong life expectancy in patients (2–6). To further improve survival outcomes, early evaluation and classification of prognosis are vital, as this can provide significant information for evaluating a patient's condition and deciding on appropriate treatments in advance. However, despite the availability of many clinical indicators such as the anion gap (AG) and serum osmolarity (7, 8), for assessing prognosis, the modest prognostic value of a single indicator and individual differences in the curative effect and toxicity of treatments make it difficult for clinicians to estimate the prognosis of CCU patients accurately and quickly.

The rapid development of medical artificial intelligence (AI) supported by big data and cloud computing makes it possible to improve the efficiency and accuracy of individual prognosis evaluation (9). AI has good adaptability in assessing disease prognosis given its abilities, including non-linear processing, high tolerance, intelligence, and self-learning. Machine learning (ML) has been widely applied in the field of disease prognosis assessment in recent years (10–13). The traditional ML models mainly include logistic regression (LR), naïve Bayes (NB), and support vector machine (SVM). Compared with serum indicators or clinical scores, these models can comprehensively evaluate patient status for accurate prognosis classification. However, these models still have many limitations. Recently, novel ML models have demonstrated improved performance compared to traditional ML models.

The eXtreme Gradient Boosting (XGBoost) model is an ML algorithm with excellent features, such as the efficient processing of missing data, flexibility, and assembly of weak prediction models to build an accurate model (14). It is an up-and-coming, widely favored algorithm in the field of ML. Besides, the establishment of specialized medical databases, such as the Medical Information Mart for Intensive Care III (MIMIC-III database), helps ML models extract data easily and enables further analysis. XGBoost (15), submitted by Tianqi Chen in 2016, is an integrated learning algorithm based on gradient boosting. It has been improved on the basis of the gradient boosting decision tree algorithm (16), with inclusion of the ability to customize the loss function, normalize the regular term, sparse feature processing, missing data processing, and parallel algorithm design, to name a few. These features allow the model to use variables with different degrees of flexibility in different areas of the output space, thereby realizing automatic feature selection and fitting of high-order interactions.

ML has made breakthroughs in the prognostic evaluation of diseases, and ML prediction models established for different diseases have achieved good prediction results. Hou et al. (17) used 4,559 sepsis patients from the MIMIC-III database and constructed XGBoost, LR, and SAPS-II score models to predict the 30-day mortality after admission in the intensive care unit (ICU). The areas under the curve (AUCs) of the three models were 0.857, 0.819, and 0.797, respectively. Li et al. (18) extracted 1,244 acute myocardial infarction (AMI) patients and built Gaussian naïve Bayes, LR, K-nearest neighbor, decision tree, random forest, and XGBoost models to predict 1-year mortality. The AUCs of the six models ranged from 0.709 to 0.942. Similarly, D'Ascenzo et al. (19) enrolled 19,826 patients diagnosed with acute coronary syndrome and constructed a risk prediction model based on ML algorithm to predict the 1-year mortality, recurrent acute myocardial infarction and bleeding risk of patients. However, most existing prognostic evaluation models use only two categories to predict the prognosis of patients, by prediction of 30-day morality and 1-year morality, which have limited clinical applications due to the lack of precision.

Therefore, we extracted CCU patients' data from the MIMIC-III database. Fifty-six clinical features were selected as inputs for the model, based on clinical experience and the completeness of prognostic indicators. Further, we attempted to construct an XGBoost model to prognosticate the time to death of CCU patients and used traditional ML models, such as LR, BN, and SVM, as benchmark comparisons. Finally, we established sub-models of each model to assess the clinical value and utility of the models. To our knowledge, this is the first study to apply a multi-category prediction approach in prognostic evaluation of CCU patients, and its findings will be of great significance to clinicians and patients.

## Materials and Methods

### Data Source

We used the MIMIC III version 1.4 for the study. MIMIC-III, an openly usable critical care database, includes data of 46,520 patients admitted to multifarious ICUs of the Beth Israel Deaconess Medical Center (BIDMC) in Boston, Massachusetts, from 2001 to 2012 (20, 21). The database contains general information (such as demographics, the dates of birth and death, ICU admission, and discharge information), laboratory parameters, vital signs, body fluid analyses, medication use, and nursing records. Permission to use the MIMIC-III database was acquired from the institutional review boards of BIDMC and the Massachusetts Institute of Technology. Moreover, the user must pass an examination to gain access to the database and be authorized by the MIMIC-III institute. Our certificate number is 9648065. All patient data from MIMIC-III were extracted using Structured Query Language (SQL).

### Study Population

**Figure 1:**
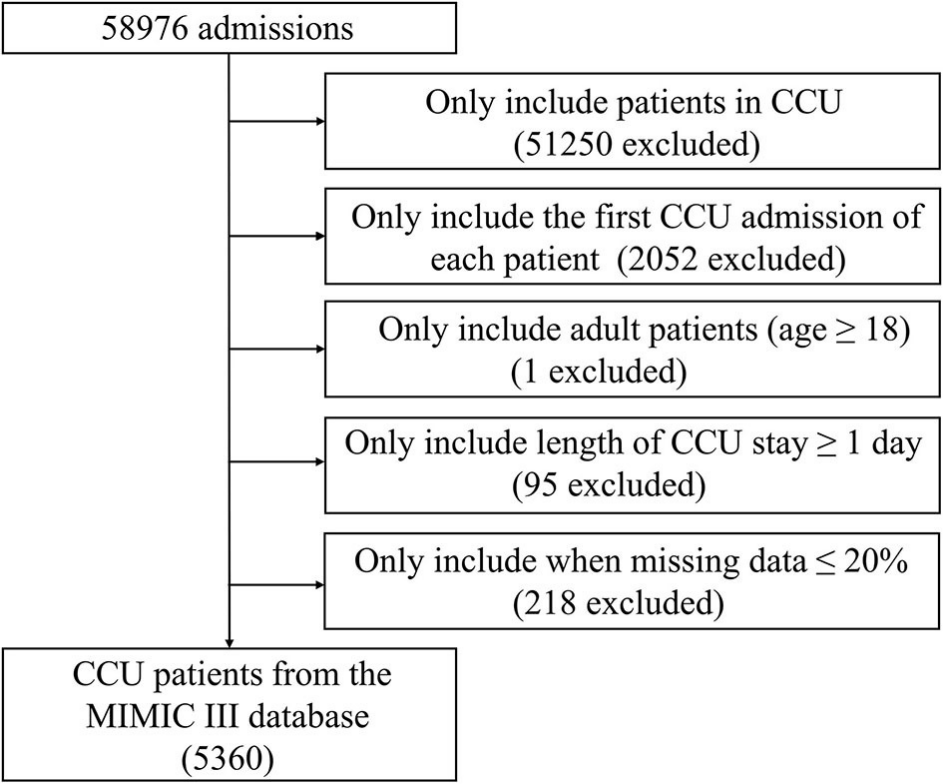
Screening flowsheet of the study population. CCU, coronary care unit; MIMIC-III, Medical Information Mart for Intensive Care III.

CCU patients registered in the MIMIC-III database were included. Only the first admission of each patient was included. The exclusion criteria were (a) age <18 years, (b) ≥20% missing individual data, and (c) length of CCU stay <1 day. Eventually, 5,360 patients were included (Figure 1).

### Data Collection

All data were extracted from the MIMIC-III database using SQL. The following data were extracted: demographics, acute physiology (vital signs and laboratory parameters), diagnoses of heart disease, comorbidities and prior myocardial infarction, medication use, and sequential organ failure score. As shown in Table 1, 56 clinical features were selected as inputs for the model, based on clinical experience and the completeness of prognostic indicators. In addition, although viral myocarditis may lead to heart failure or cardiac arrest (22), this indicator was excluded because of the lack of sufficient samples in the MIMIC-III database. Serum osmolarity was calculated using the equation (2 × Na^+^ + K^+^) + (glucose/18) + (urea nitrogen/2.8) (7). Only values of the three variables measured at the same time were used for calculations. All laboratory parameters and vital signs were extracted within 24 h of CCU admission; we calculated the mean of each indicator separately.

**Table 1:** Predictor variables used in this study.

**Predictor**	
**Demographic data**	Serum osmolarity^*^
Age	Urine output^*^
Gender	**Diagnosis of heart disease**
Ethnicity	Coronary heart disease (CHD)
Height	Acute myocardial infarction (AMI)
Weight	AMI_ anterior wall
Body mass index (BMI)	Atrial fibrillation
**Acute physiology (first 24 h in the CCU)**	Ventricular arrhythmias^†^
**Vital signs**	Third-degree atrioventricular block
Heart rate^*^ (HR)	Congestive heart failure (CHF)
Systolic blood pressure^*^ (SBP)	Primary cardiomyopathy^‡^
Diastolic blood pressure^*^ (DBP)	Valve disease
Mean blood pressure^*^(MAP)	Endocarditis
Respiratory rate^*^ (RR)	Cardiogenic shock
Temperature^*^ (TEMP)	**Comorbidity and medical history**
blood oxygen saturation^*^ (SpO2)	Diabetes
**Laboratory parameters**	Chronic obstructive pulmonary diseases (COPD)
Anion gap^*^ (AG)	Hypertension
Bicarbonate^*^	Respiratory failure
Glucose^*^	Hypercholesterolemia
Sodium^*^	Chronic liver disease
Potassium^*^	Chronic kidney disease
Calcium^*^	Prior myocardial infarction
Chloride^*^	**Medication use**
Creatinine^*^	Antiplatelet
Blood urea nitrogen^*^ (BUN)	Anticoagulants
White blood cell^*^ (WBC)	Beta-blocks
Hemoglobin^*^	ACEI/ARB
Platelet^*^	Statin
Mean corpuscular volume^*^ (MCV)	Vasopressin
Mean corpuscular hemoglobin^*^ (MCH)	**Other**
Red blood cell volume distribution width^*^ (RDW)	Sequential organ failure score (SOFA)

* Each predictor marked with * means that it is a time-stamped variable, and its corresponding average values within the first 24 h in the CCU were used as inputs in model development.

† *Includes ventricular tachycardia, ventricular flutter, and ventricular fibrillation*.

‡ *Includes disorders of mitral, aortic, pulmonary, and tricuspid valve; rheumatic diseases of valves and congenital diseases of valve. CCU, coronary care unit; ACEI/ARB, angiotensin-converting enzyme inhibitor/angiotensin receptor blocker*.

### Outcome and Statistical Analysis

The outcome was time to death, defined as the time from CCU admission to death. Fatality information was extracted from the file named “Patients” in the MIMIC-III database. Based on the time to death, we divided patients in this study into four groups: <30 days (class 0), 30 days−1 year (class 1), 1–5 years (class 2), ≥5 years (class 3), and variables were displayed and compared between the groups. Normally and non-normally distributed continuous variables were, respectively, summarized as the mean ± SD and the median (interquartile ranges, IQR). One-way analysis of variance or the Kruskal-Wallis test was used to analyse differences. Categorical variables were summarized as a number (percentage) and were compared between groups using the chi-square test or Fisher' exact test. All analyses were performed using the STATA 15 software, and statistical difference was defined as *p*-value < 0.05.

### Model and Metrics

In the model-construction phase, we employed an ML model using XGBoost to predict the time to death, while using LR, NB, and SVM models as benchmark comparisons. For XGBoost, we set the reduction rate to 0.3, the maximum tree depth as 2, while other parameters were set to the default parameters of the scikit-learn library. In the model-comparison phase, we tested and compared the performances of the four predictive models for their accuracy (ACC), F1 score, Matthews correlation coefficient (MCC), and AUCs of the receiver operating characteristic curves (ROC). The AUCs and F1 score were calculated by micro-average and macro-average methods (23). For classification tasks with imbalanced data, AUCs, the F1 score, and MCC have better adaptability (24). Thus, these three indicators were included in the performance evaluation of the model. Subsequently, we performed the Local Interpretable Model-Agnostic Explanations (LIME) algorithm to obtain the direction in which the features change. LIME places emphasis on training local surrogate models to explain individual predictions (25). Besides, to further assess the clinical practicability of the model, we divided patients into four two-class data sets according to the time of death (for example, class 1 is for one group, classes 0, 2, and 3 are for another group) and sequentially established sub-models of each model. Decision curve analysis (DCA) was used to calculate the net benefit and compare differences between these four sub-models. The optimal model was further analyzed using a clinical impact curve (CIC) to assess the clinical practicability and net benefit of the model with the best prognostic predictive value. Finally, the feature ablation curves (excluded one by one according to the feature importance score from low to high) of the XGBoost model were conducted to obtain the simplified model.

For all the models above, we used a 10-fold cross validation method to obtain the performance of the model for the whole data set. For cross validation, the dataset was divided into 10-folds, of which 1-fold was used as the test set and the remaining were used as the training set; all results of the 10 repetitions were averaged as the overall performance. In the XGBoost model, we used 20% of the training set as the validation set to perform the early stopping strategy. All experiments of the XGBoost model and other models were constructed using the scikit-learn of the standard ML software package in the Python 3.8 software.

## Results

### Baseline Characteristics

In total, 5,360 patients admitted to the CCU were included (Figure 1). The baseline characteristics of patients stratified by time to death are displayed in Supplementary Table S1. Except for the ethnicity, third-degree atrioventricular block, primary cardiomyopathy, chronic liver disease, prior myocardial infarction, and blood oxygen saturation (SpO_2_), other clinical features showed a statistically significant difference between the groups (*p* < 0.05).

### Model Comparisons and Validations

**Figure 2:**
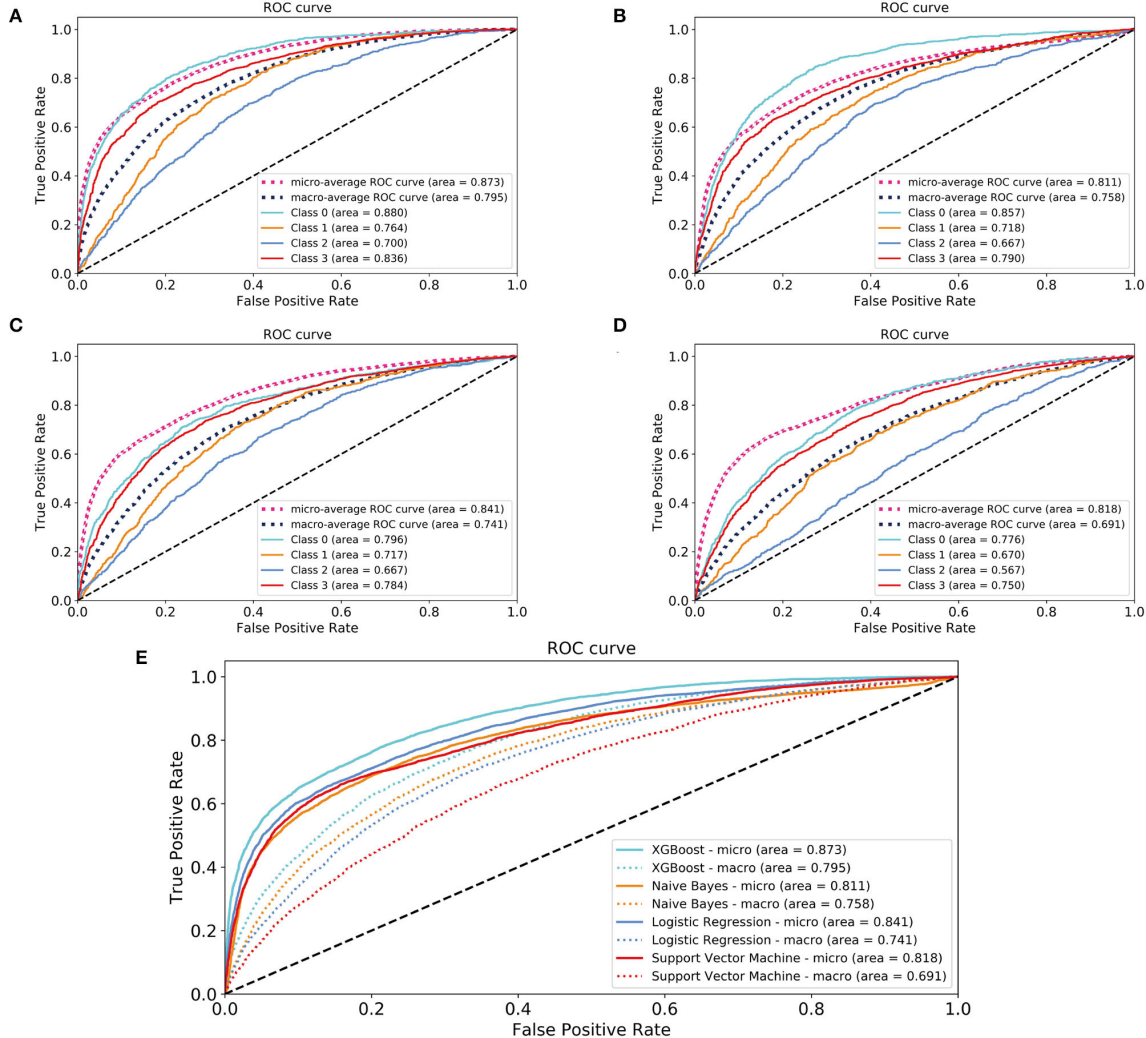
Areas under the receiver operating characteristic curves for evaluating the classification performance of the different models. **(A)** XGBoost model; **(B)** naïve Bayes model; **(C)** logistic regression model; **(D)** support vector machine model; **(E)** a comparison of four models. Class 0: time to death <30 days; Class 1: 30 days ≤ time to death <1 year; Class 2: 1 year ≤ time to death <5 years; Class 3: time to death ≥5 years. XGBoost, eXtreme Gradient Boosting.

In the model-construction and validation phase, ML models had different recognition and classification capabilities for different classes. These capabilities had some consistency, that is, the model had better classification capabilities for classes 0 and 3, while the classification performance for classes 1 and 2 was poor. If the XGBoost model is taken as an example, the micro-AUCs of classes 0 and 3 were 0.88 and 0.836, respectively; those of classes 1 and 2 were 0.764 and 0.7, respectively (Figures 2A–D). All four models (XGBoost model, NB model, LR model, and SVM model) showed good discriminatory power with micro-AUCs of 0.873 (95% CI 0.867–0.879), 0.811 (95% CI 0.802–0.820), 0.841 (95% CI 0.833–0.849), and 0.818 (95% CI 0.812–0.825), respectively, and macro-AUCs of 0.795 (95% CI 0.782–0.808), 0.758 (95% CI 0.745–0.772), 0.741 (95% CI 0.727–0.756), and 0.691 (95% CI 0.678–0.711), respectively. The accuracy and F1-micro of the models were 0.663 (95% CI 0.655–0.671), 0.605 (95% CI 0.594–0.617), 0.632 (95% CI 0.621–0.642), and 0.622 (95% CI 0.618–0.627), respectively. The MCCs of models were 0.337 (95% CI 0.318–0.357), 0.317 (95% CI 0.295–0.340), 0.250 (95% CI 0.224–0.276), and 0.182 (95% CI 0.166–0.198), respectively. These indicators showed that the XGBoost model was the most optimal option, although its F1-macro was not the largest among the four models (Figure 2E; Table 2).

**Table 2:** Performance of the four prediction models.

	**XGBoost**	**NB**	**LR**	**SVM**
Accuracy (%), 95% CI	0.663 (0.655–0.671)	0.605 (0.594–0.617)	0.632 (0.621–0.642)	0.622 (0.618–0.627)
AUC-micro, 95% CI	0.873 (0.867–0.879)	0.811 (0.802–0.820)	0.841 (0.833–0.849)	0.818 (0.812–0.825)
AUC-macro, 95% CI	0.795 (0.782–0.808)	0.758 (0.745–0.772)	0.741 (0.727–0.756)	0.691 (0.678–0.711)
F1-micro, 95% CI	0.663 (0.655–0.671)	0.605 (0.594–0.617)	0.632 (0.621–0.642)	0.622 (0.618–0.627)
F1-macro, 95% CI	0.416 (0.395–0.434)	0.441 (0.424–0.455)	0.343 (0.326–0.360)	0.275 (0.262–0.287)
MCC, 95% CI	0.337 (0.318–0.357)	0.317 (0.295–0.340)	0.250 (0.224–0.276)	0.182 (0.166–0.198)

*NB, Naive Bayes; LR, logistic regression; SVM, support vector machine; CI, confidence interval; AUC, area under the curve; MCC, Matthews correlation coefficient*.

The performance indicators of the validation set and test set under the 10-fold cross validation test of the XGBoost model are shown in Table 3. There was no significant difference between the two sets. Subsequently, we applied the XGBoost model to the subgroup analysis of the four major heart diseases. The results are shown in Table 4, and the models all showed good performance. The coronary heart disease subgroup was the best.

**Table 3:** Performance of the validation set and test set under the 10-fold cross-validation test of the XGBoost model.

	**Validation set**	**Test set**
Accuracy, 95% CI	0.661 (0.654–0.667)	0.663 (0.655–0.671)
AUC-micro, 95% CI	0.870 (0.867–0.874)	0.873 (0.867–0.879)
AUC-macro, 95% CI	0.789 (0.783–0.795)	0.795 (0.782–0.808)
F1-micro, 95% CI	0.661 (0.654–0.667)	0.663 (0.655–0.671)
F1-macro, 95% CI	0.420 (0.410–0.430)	0.416 (0.395–0.434)
MCC, 95% CI	0.336 (0.320–0.352)	0.337 (0.318–0.357)

*CI, confidence interval; AUC, area under the curve; MCC, Matthews correlation coefficient*.

**Table 4:** Performance of the XGBoost model in the four major types of heart disease.

	**CHD**	**AMI**	**CHF**	**VA**
Accuracy (%), 95% CI	0.703 (0.691–0.715)	0.751 (0.725–0.777)	0.571 (0.551–0.592)	0.666 (0.628–0.704)
AUC-micro, 95% CI	0.897 (0.888–0.906)	0.917 (0.903–0.931)	0.811 (0.797–0.826)	0.873 (0.855–0.891)
AUC-macro, 95% CI	0.812 (0.791–0.834)	0.815 (0.782–0.849)	0.755 (0.733–0.777)	0.806 (0.773–0.838)
F1-micro, 95% CI	0.703 (0.691–0.715)	0.751 (0.725–0.777)	0.571 (0.551–0.592)	0.666 (0.628–0.704)
F1-macro, 95% CI	0.428 (0.401–0.456)	0.394 (0.348–0.439)	0.414 (0.392–0.435)	0.409 (0.357–0.461)
MCC, 95% CI	0.343 (0.310–0.375)	0.371 (0.294–0.448)	0.306 (0.279–0.333)	0.384 (0.310–0.458)

*CHD, coronary heart disease; AMI, acute myocardial infarction; CHF, congestive heart failure; VA, ventricular arrhythmia; CI, confidence interval; AUC, area under the curve; MCC, Matthews correlation coefficient*.

### Features Assessed Using XGBoost

**Figure 3:**
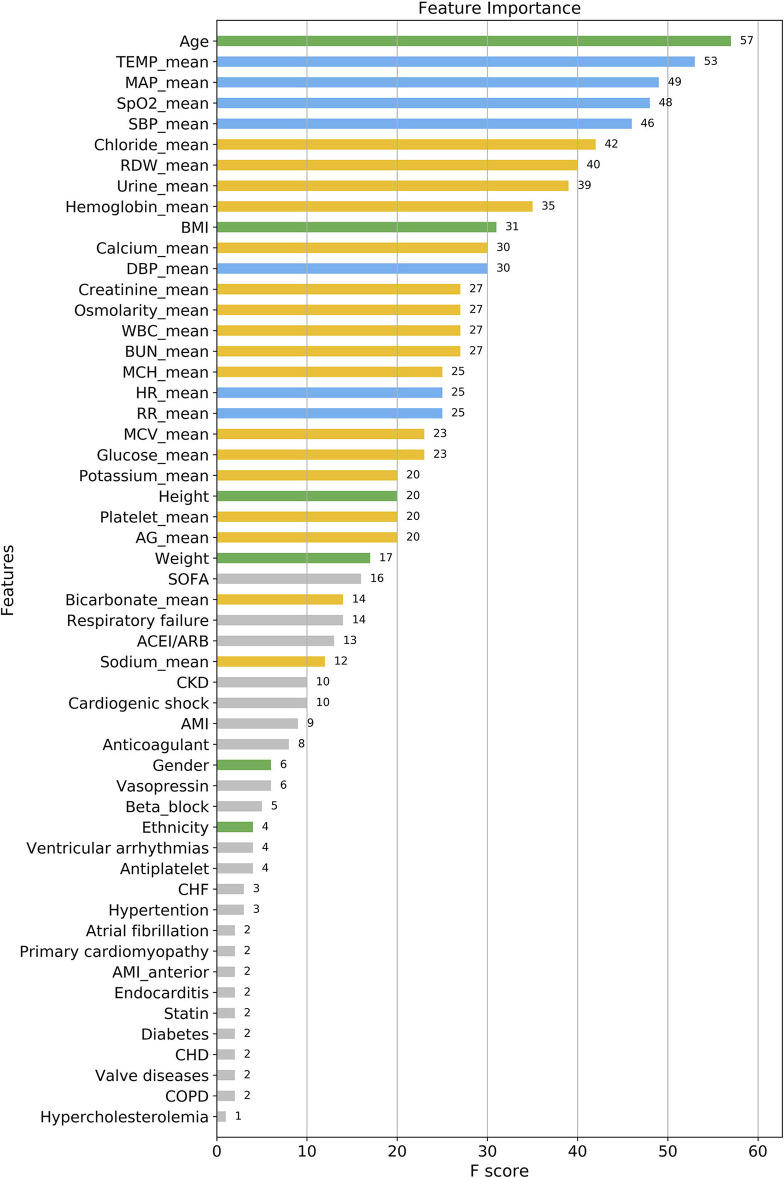
Feature importance score ranking for 56 clinical features of the four-group XGBoost predictor. The information reflects the contribution of different features to the classification performance of XGBoost model (from top to bottom). **Green**, demographic data; **Blue**, vital signs; **Yellow**, laboratory parameters; **Gray**, others. TEMP, temperature; MAP, mean arterial pressure; SpO2, oxygen saturation; SBP, systolic blood pressure; RDW, red blood cell volume distribution width; BMI, body mass index; DBP, diastolic blood pressure; WBC, white blood cell; BUN, blood urea nitrogen; MCH, mean corpuscular hemoglobin; HR, heart rate; RR, respiratory rate; MCV, mean corpuscular volume; AG, anion gap; SOFA, sequential organ failure score; CKD, chronic kidney disease; AMI, acute myocardial infarction; CHF, congestive heart failure; CHD, coronary heart disease; COPD, chronic obstructive pulmonary diseases.

As shown in Figure 3, according to the results of each feature's analysis in the XGBoost model, age was most important feature of the data set. The remaining top 10 features were temperature, mean arterial pressure (MAP), SpO_2_, systolic blood pressure (SBP), chloride, red blood cell volume distribution width urine_24 h, hemoglobin, and body mass index, in that order. However, traditional prognostic-related indicators, such as diabetes and hypercholesterolemia, showed poor importance contribution scores.

### Interpretability of the Prediction Model

**Figure 4:**
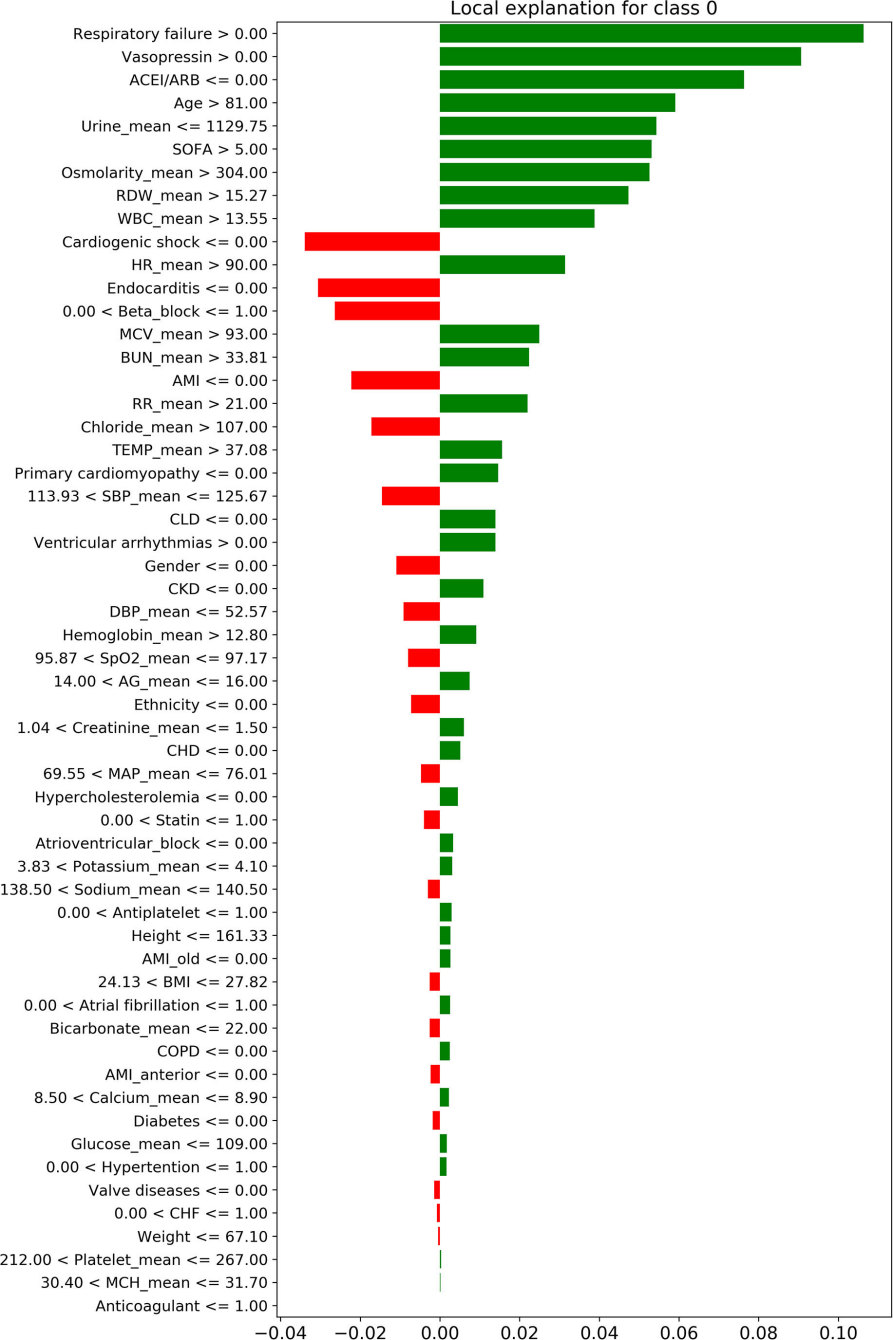
The local interpretation of the XGBoost model for class 0. The features of the green column make the model identify the sample as class 0, while the features of the red column allow the model to identify the sample as non-class 0. Since the sum of green column score exceeded red, the model finally identifies this sample as class 0.

Figure 4 shows the decision process for the single-sample prediction of class 0, which is a local interpretation of the XGBoost model based on LIME. This sample was correctly classified as class 0 by the model, where the features in green allowed the model to identify the sample as class 0, and the features in red allowed the model to identify the sample as not class 0. The LIME results of classes 1–3 are shown in Supplementary Figure S1.

### Sub-models Comparisons

**Figure 5:**
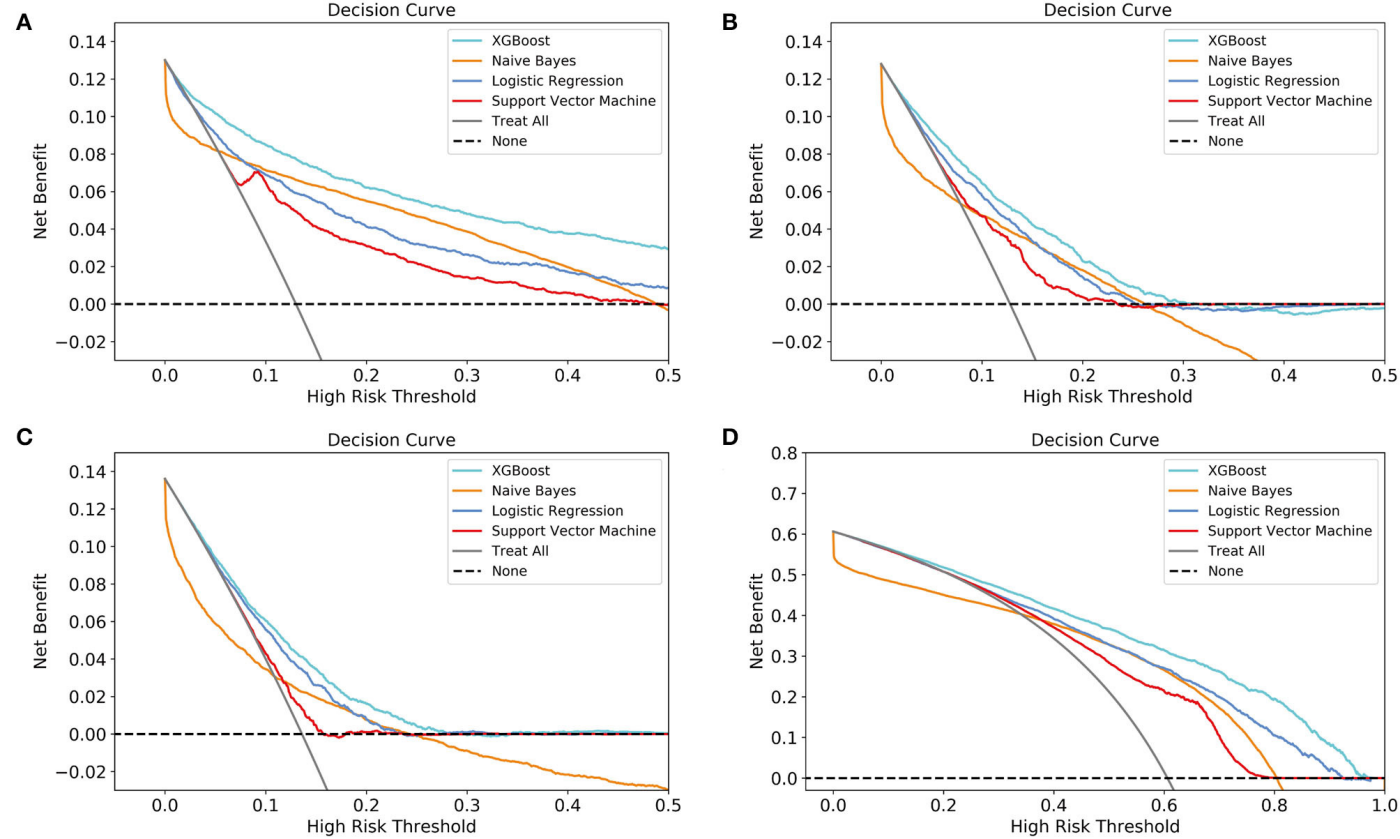
Decision curve analysis (DCA) of the four prediction sub-models. **(A)** class 0 (<30 days); **(B)** class 1 (30 days−1 year); **(C)** class 2 (1–5 years); **(D)** class 3 (≥5 years). The net benefit curves for the four prognostic sub-models are shown. The lateral-axis shows the threshold probability for different class outcomes, and the direct-axis shows the net benefit. The horizontal dashed line represents no intervention in all patients, with a net benefit of 0, and the sloping gray line represents intervention in all patients. The four colored curves represent the four schemes (prediction models) with a larger net benefit to XGBoost compared to the other models for the threshold probabilities of different outcomes.

**Figure 6:**
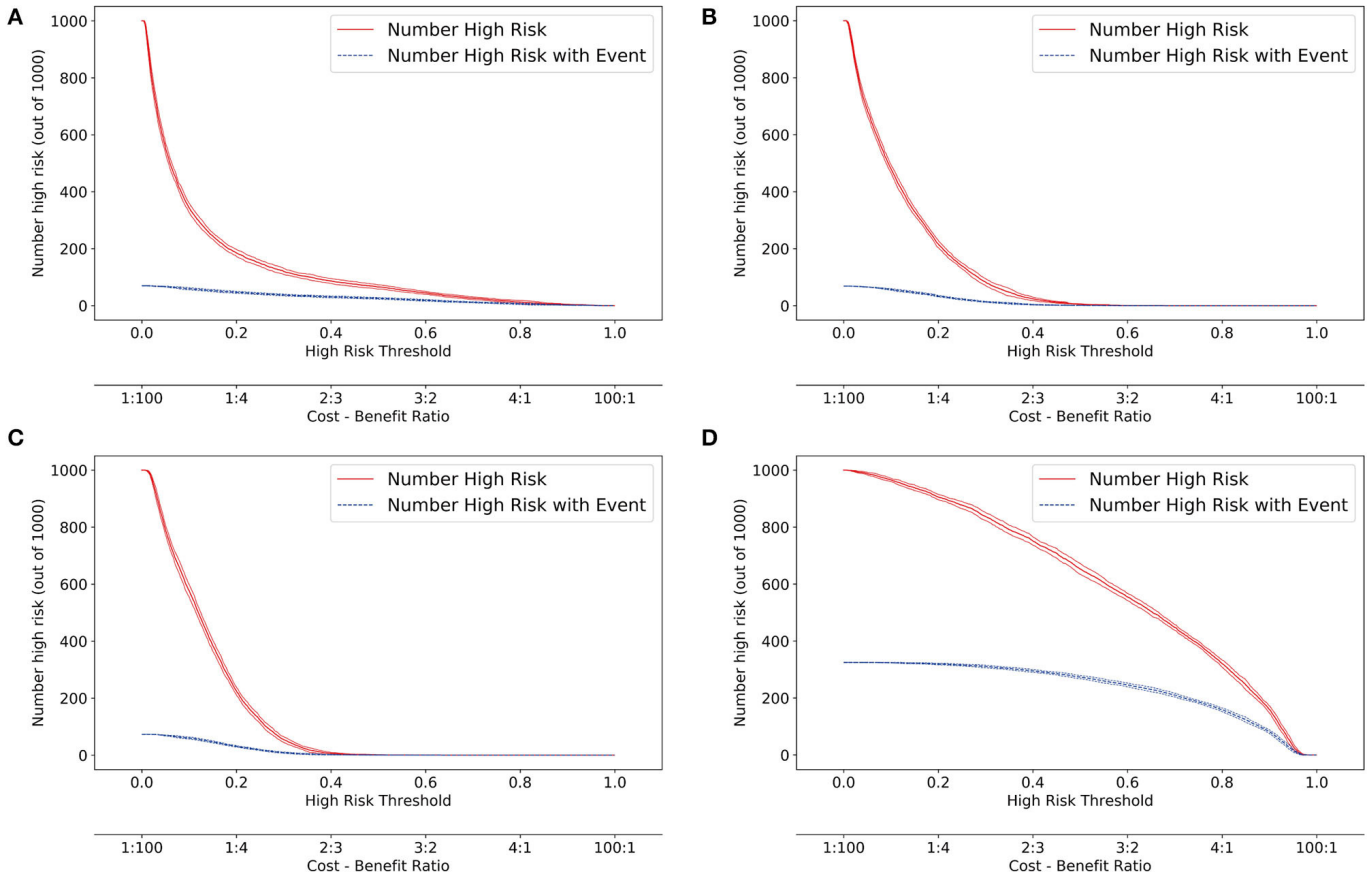
Clinical impact curve (CIC) of the XGBoost model. **(A)** class 0 (<30 days); **(B)** class 1 (30 days−1 year); **(C)** class 2 (1–5 years); **(D)** class 3 (≥5 years). The red curve (number of high-risk individuals) indicates the number of people classified as positive (high risk) by the model at each threshold probability; the blue curve (number of high-risk individuals with outcome) is the number of true positives at each threshold probability.

According to DCA of four prediction sub-models, the net benefit for the XGBoost model was all greater than that of the traditional models for the threshold probabilities of different outcomes, meaning that the XGBoost model was the most optimal (Figures 5A–D). Thus, the XGBoost model was further analyzed using CIC. The CIC is shown in Figures 6A–D, and clearly shows that the XGBoost model had an excellent clinical net benefit within the general range of threshold probabilities and impacted patient outcomes, which verifies that the XGBoost model had better clinical decision-making performance than the other models for different categories of patients. Table 5 shows the quantitative results of the DCA and CIC curves of the XGBoost sub-model. For example, for class 0, the risk probability threshold of 0.15 (cost-benefit ratio 15:85) corresponds to at least 75% of the population. This means that <25% of patients were classified as positive by the model (245 patients). Among these, 99 patients had a positive outcome. The calculated net benefit is as follows: 99/1,000–(245–99)/1,000 × 0.15/(1–0.15) = 0.07.

**Table 5:** Critical prediction accuracy under different XGBoost sub-model risk thresholds.

**Model risk percentile**	**RPT**	**Cost-benefit ratio**	**NHR (out of 1,000)**	**NHR with event (out of 1,000)**	**Sensitivity (%)**	**Specificity (%)**	**NB**
**<30 days (class 0)**							
≥0	0.00	1: ∞	1,000	130	100	0	0.13
≥25	0.03	3: 97	710	127	97	33	0.11
≥50	0.06	6: 94	497	121	93	57	0.10
≥75	0.15	15: 85	245	99	76	83	0.07
≥90	0.36	36: 64	99	62	48	96	0.04
**30 days−1 year (class 1)**							
≥0	0.00	1: ∞	1,000	128	100	0	0.13
≥25	0.04	4: 96	738	124	97	30	0.10
≥50	0.10	1: 9	480	105	83	57	0.06
≥75	0.19	19: 81	239	70	55	81	0.03
≥90	0.29	29: 71	91	28	22	93	0.00
**1–5 years (class 2)**							
≥0	0.00	1: ∞	1,000	136	100	0	0.14
≥25	0.06	6: 94	740	126	93	29	0.09
≥50	0.12	12: 88	498	103	76	54	0.05
≥75	0.20	20: 80	225	58	43	81	0.02
≥90	0.27	27: 73	90	26	19	93	0.00
**≥5 years (class 3)**							
≥0	0.00	1: ∞	1,000	606	100	0	0.61
≥25	0.40	2: 3	750	550	91	49	0.42
≥50	0.66	66: 34	494	422	70	82	0.28
≥75	0.85	85: 15	246	230	38	96	0.14
≥90	0.93	93: 7	84	81	13	99	0.04

*RPT, risk probability thresholds; NHR, number high risk; NB, net benefit*.

### Simplified Model

**Figure 7:**
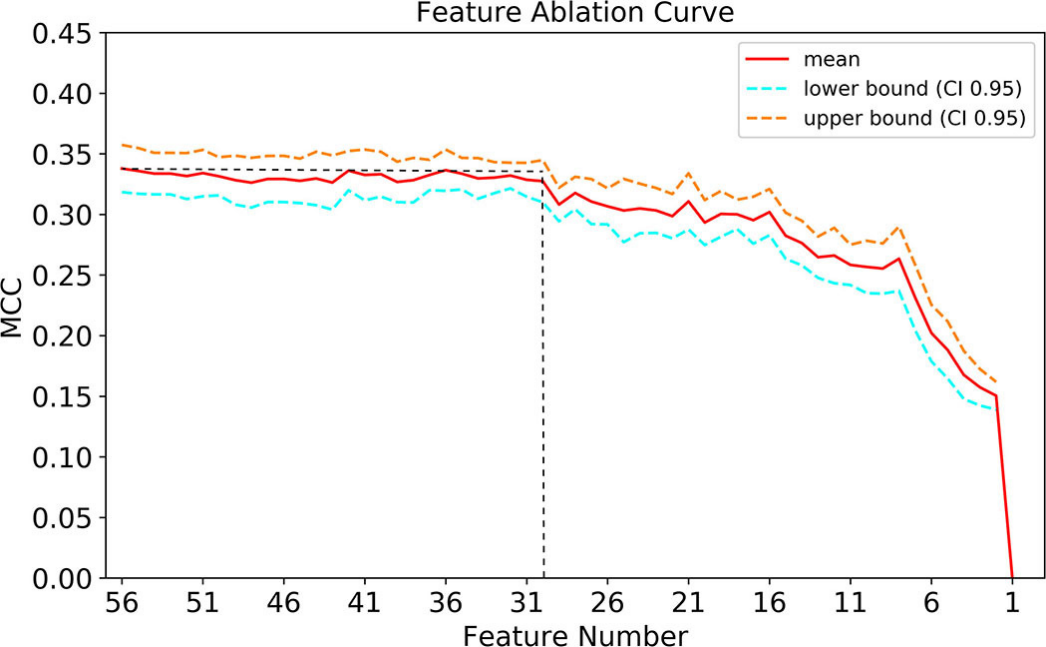
Feature ablation curves of XGBoost model. MCC, Matthews correlation coefficient; CI, confidence interval.

As presented in Figure 7, When the number of features of the XGBoost model is reduced from 56 to 30 one by one, the MCC remains basically constant, that is, TOP30 in Figure 3 is the input feature of the simplified model. The detailed information of Figure 7 is listed in Supplementary Table S2.

## Discussion

In this study based on the need for clinical applications, we pioneered the multi-category ML model for predicting time to death, rather than the traditional two-category model, for the first time. We found that the XGBoost model, when compared with some traditional classification models, showed obvious superiority in classification performance and clinical utility for different categories of patients.

In previous ML model studies, predictive performance was evaluated and compared. However, clinical applicability and clinical consequences were not investigated. These models, including XGBoost (17, 18, 26, 27), were applied to patients diagnosed with AMI, those who underwent open-heart surgery, or those admitted to the ICU. All of them had a two-category pattern, that is, they focused on identifying high-risk populations for early intervention. However, this type of model is limited by the fact that patients outside the high-risk category are not accurately classified. Therefore, some intermediate-risk groups of patients do not receive clinicians' full attention. In our study, we divided patients into four categories according to the time to death. We further optimized the XGBoost micro-parameters, making it more suitable for multi-category prediction. Consequently, our ML model could assist CCU physicians in developing treatment strategies and determining the follow-up intensity according to different risk levels. For example, when patients enter the CCU, their baseline data, vital signs, and laboratory data on the first day will be inputted into the model for analysis. According to the analysis results, the patient's prognosis can be stratified. Those predicted to die within 30 days are classified in the high-risk group. Improved vital sign monitoring and continue hospitalization are recommended for such patients. Those predicted to die between 30 days and 1 year belong to the medium-risk group. Increased follow-up frequency after discharge is recommended, and the attending physician should pay attention to these patients' potential risks. Those predicted to die 1 year later are categorized as the low-risk group and should be followed up regularly after discharge. However, these are only approximate clinical decisions. Moreover, specific treatment measures also depend on the immediate state of the individual. This study has not discussed this in depth.

An important finding of this study was that the ML model had a different classification performance for different classes, but still had a few commonalities. All models had better classification capabilities for classes 0 and 3, and classification performance for classes 1 and 2 was poor. This phenomenon may be explained as follows. First, the grouped data set was unbalanced. More than 3,000 patients survived for more than 5 years. The models we built were all supervised ML models. When the samples are unbalanced, the model tends to ignore the small sample loss to reduce the overall loss (28). Due to the model's intrinsic characteristics, the classification results are often influenced several categories, resulting in the overestimation of classification performance. Therefore, the model had a higher classification accuracy for survival over 5 years. Further, the scales of classes 0, 1, and 2 were very similar and they all had about 700 patients. This may have attributed to the immediacy of the clinical indicators. The patients' physiological state changes all the time. Over time, the predictive performance of the indicator collected early may decline. Three studies published previously, in fact, have predicted the short-term prognosis of patients based on this feature (17, 18, 26).

Additionally, many studies applied statistical methods to initially screen predictors and then, incorporated the screened factors into the model. However, we did not pre-process the input factors for the following reasons: firstly, in the initial selection process, we screened out these 56 predictors from hundreds of clinical factors in the database based on the literature and clinical practical applications. Secondly, for the first time, we innovatively divided the patient's time to death into multiple intervals for prediction. We do not know whether predictors with or without significant differences in traditional two-category studies are applicable to multi-category situations. Furthermore, traditional statistical screening methods may have limitations in case of multiple classifications. This may have led to over-screening or meaningless screening of predictors. Finally, the XGBOOST algorithm model used can automatically screen the importance of predictive variables while ignoring the interference of irrelevant variables, which greatly improved the effectiveness of our research.

The AUCs, accuracy, F1 score, and MCC testified for the excellent performance of the XGBoost model. The XGBoost model builds a host of sub-models for classification, and finally assembles the classification results. Since the sub-model only uses a few indicators for model construction, some of the outliers and missing values will have a smaller impact on the performance of the model, thus making the model more robust (15). This feature has good suitability for the MIMIC-III database. Moreover, the XGBoost algorithm can standardize the regularization term to prevent the model from overfitting. Thus, these features enable the model to have a stronger classification performance for retrospective data. However, accuracy, AUCs, and the F1 score focus solely on the predictive accuracy of the model, without the results caused by the prediction information. For improvement in purely mathematical metrics, DCA is widely used in clinical analysis (17, 29). DCA is based on a decision-making theoretical framework that considers both the benefit of the intervention and the cost of the intervention for patients who cannot benefit (30). Therefore, DCA can compare the clinical application value of different models and tell us which model is worth using. However, DCA is used to evaluate the clinical value of the two-category model. To make it suitable for multi-category models, we divided the patients into binary data sets in turn, for example, group 1: class 2 and group 2: classes 0, 1, and 3, and built sub-model of each model. Then, DCA was used to evaluate the clinical practicability and decision-making performance of different models for patients with different outcomes.

The relationship between the contribution features of the XGBoost model and death cannot be fully explained. Thus, further research is needed to investigate the specific relationship between these features and death. The following is a brief summary of the important results obtained by the XGBoost model. Among these features, the weight of age was the greatest, meaning that it was the most significant predictor for the time to death of CCU patients. This result is consistent with those of previous clinical studies. Albanese et al. (31) reported that for CCU patients after percutaneous coronary intervention, older age was associated with major endpoints such as ventricular fibrillation, tachycardia, and sudden cardiac or arrhythmic death. Al-Ghamdi et al. (32) concluded that age >50 years was an independent predictor of death in CCU patients. Ruiz-Bailén et al. (33) enrolled 17 761 CCU/ICU patients with AMI, and indicated that age was an important independent predictive variable for mortality. This may be due to the following potential mechanisms: first, older patients tend to have more complications and infection risks (33); second, older patients, despite the higher mortality risk, are treated with less aggressive therapies than younger patients (34); finally, older patients show poor adaptability and tolerance under stressful conditions such as hypoxia, myocardial ischaemia, and so on. Besides, we find that the top 2–4 important features of the XGBoost model are temperature, MAP, and SpO_2_, which are all clinically vital signs. This reminds clinicians to focus on the modest change in patients' vital signs at an early stage. Vital signs have been shown to be the most accurate predictors of clinical deterioration (35). In the CCU, hyperthermia often indicates infection and hypothermia indicates shock, both of which are predictors of poor prognosis; body temperature is thus a good prognostic factor. Similarly, MAP, SBP, and SpO_2_ reflect the respiratory and circulatory state of the patients, and their abnormalities may indicate early physiological duress. However, traditional prognostic indicators, such as diabetes, hypercholesterolemia, had poor contribution scores. On one hand, these indicators may display lower performance in predicting death in multi-class classification. In contrast, due to them being categorical variables, the model may reduce its prognostic classification weight while simultaneously dealing with categorical and continuous data.

Our study has several limitations due to its retrospective design. First, a few patients had small amounts of missing data. Although statistical methods were used to compensate, they could also have led to data bias and inaccurate prediction results. Second, measurement bias within calculations is possible, as the methods were based on specialists' individual opinion. Finally, as patient data were extracted from the MIMIC-III database, clinically common prognostic indicators of cardiovascular disease, such as troponin, creatine kinase-MB, and lactate, were excluded because the measurement volume was too small. Nonetheless, the XGBoost model is an efficient and robust method for multi-categorically predicting patients' time to death.

## Conclusions

In summary, our study indicates that the XGBoost model does outperform traditional models. It has the potential to assist physicians in the CCU to perform optimal clinical interventions quickly and accurately, and may thus improve the prognosis of CCU patients.

## Data Availability Statement

The original contributions presented in the study are included in the article/Supplementary Material, further inquiries can be directed to the corresponding author/s.

## Author Contributions

YW, XizW, LZ, DZ, JunZ, HH, and SW collected and interpreted the data. XinW, TZ, and YL analyzed the data. XinW, TZ, and MX generated the figures and wrote the manuscript. DL and JunhZ designed study and revised the manuscript. All authors approved the final manuscript.

## Funding

This work was supported by grants from the National Natural Science Foundation of China (Nos. 81974025 and 81941003), Natural Science Foundation of Zhejiang Province (Nos. LY19H020007 and LY18H020001), and Medical and Health Science Program of Zhejiang Province (Nos. 2020RC016 and 2019RC183).

## Conflict of Interest

The authors declare that the research was conducted in the absence of any commercial or financial relationships that could be construed as a potential conflict of interest.

## Publisher's Note

All claims expressed in this article are solely those of the authors and do not necessarily represent those of their affiliated organizations, or those of the publisher, the editors and the reviewers. Any product that may be evaluated in this article, or claim that may be made by its manufacturer, is not guaranteed or endorsed by the publisher.
