# Supplementary material for: Predicting the Prognosis of Patients in the Coronary Care Unit: A Novel Multi-Category Machine Learning Model Using XGBoost
Source: Front Cardiovasc Med. 2022 May 12;9:764629. doi: 10.3389/fcvm.2022.764629 (PMC9133425; doi:10.3389/fcvm.2022.764629)
Supplement: Supplementary file 1 [file Data_Sheet_1.zip › Supplementary Materials.docx]

**Supplementary Table S1** Baseline characteristics of mimic-III patients in CCU

|  | **Total** | **Death < 30days** | **30days ≤ Death < 1year** | **1year ≤ Death < 5year** | **Death ≥ 5years** | **P** |
| --- | --- | --- | --- | --- | --- | --- |
| Number (sample size) | 5360 | 697 | 686 | 729 | 3248 |  |
| Age (year) | 71(58-81) | 77(66-85) | 78(68-85) | 76(67-83) | 66(55-77) | <0.0001 |
| Gender n (%) |  |  |  |  |  |  |
| Male  Female | 3127(58.34)  2233(41.66) | 366(52.51)  331(47.49) | 371(54.08)  315(45.92) | 409(56.10)  320(43.90) | 1981(60.99)  1267(39.01) | <0.0001 |
| Ethnicity n (%) |  |  |  |  |  |  |
| White  Black  Yellow  Others | 3751(69.98)  342(6.38)  84(1.57)  1183(22.07) | 470(67.43)  37(5.31)  9(1.29)  181(25.97) | 476(69.39)  54(7.87)  15(2.19)  141(20.55) | 527(72.29)  39(5.35)  6(0.82)  157(21.54) | 2278(70.14)  212(6.53)  54(1.66)  704(21.67) | 0.051 |
| Weight (kg) | 80(67.1-95) | 75.1(63-90) | 73.1(61-88.8) | 78.2(64.4-92) | 81.9(70.3-97.05) | <0.0001 |
| Height (cm) | 169.16±10.54 | 167.47±10.34 | 167±10.19 | 168.17±10.62 | 170.21±10.5 | <0.0001 |
| BMI (kg/m2 ) | 27.82(24.13-32.01) | 26.55(22.82-30.6) | 26.17(22.42-30.45) | 27.15(23.6-31.55) | 28.11(24.84-32.6) | <0.0001 |
| SOFA | 3(1-5) | 6(3-9) | 4(2-6) | 3(2-5) | 2(1-4) | <0.0001 |
| Diagnoses of heart disease n (%) |  |  |  |  |  |  |
| Coronary heart disease | 2987(55.73) | 289(41.46) | 337(49.13) | 408(55.97) | 1953(60.13) | <0.0001 |
| Acute myocardial infarction  AMI_ anterior wall | 1232(22.99)  485(9.05) | 159(22.81)  66(9.47) | 87(12.68)  37(5.39) | 122(16.74)  42(5.76) | 864(26.60)  340(10.47) | <0.0001 |
| Atrial fibrillation | 1630(30.41) | 284(40.75) | 283(41.25) | 251(34.43) | 812(25.00) | <0.0001 |
| Ventricular arrhythmias | 768(14.33) | 145(20.80) | 89(12.97) | 93(12.76) | 441(13.58) | <0.0001 |
| Third-degree atrioventricular block | 240(4.48) | 28(4.02) | 28(4.08) | 36(4.94) | 148(4.56) | 0.8 |
| Congestive heart failure | 2285(42.63) | 360(51.65) | 397(57.87) | 391(53.64) | 1137(35.01) | <0.0001 |
| Primary cardiomyopathy | 379(7.07) | 38(5.45) | 60(8.75) | 49(6.72) | 232(7.14) | 0.118 |
| Valve disease | 980(18.28) | 133(19.08) | 177(25.80) | 152(20.85) | 518(15.95) | <0.0001 |
| Endocarditis | 59(1.10) | 15(2.15) | 8(1.17) | 5(0.69) | 31(0.95) | 0.031 |
| Cardiogenic shock | 499(9.31) | 186(26.69) | 66(9.62) | 38(5.21) | 209(6.43) | <0.0001 |
| Comorbidities and medical history n (%) |  |  |  |  |  |  |
| Diabetes | 1622(30.26) | 207(29.70) | 228(33.24) | 264(36.21) | 923(28.42) | <0.0001 |
| COPD | 114(2.13) | 25(3.59) | 25(3.64) | 28(3.84) | 36(1.11) | <0.0001 |
| Hypertension | 2432(45.37) | 251(36.01) | 226(32.94) | 326(44.72) | 1629(50.15) | <0.0001 |
| Respiratory failure | 687(12.82) | 244(35.01) | 132(19.24) | 78(10.70) | 233(7.17) | <0.0001 |
| Hypercholesterolemia | 777(14.50) | 58(8.32) | 68(9.91) | 113(15.50) | 536(16.50) | <0.0001 |
| Chronic liver disease | 20(0.37) | 2(0.29) | 1(0.15) | 5(0.69) | 12(0.37) | 0.451 |
| Chronic kidney disease | 602(11.23) | 95(13.63) | 125(18.22) | 79(10.84) | 303(9.33) | <0.0001 |
| Prior myocardial infarction | 474(8.84) | 47(6.74) | 47(6.85) | 72(9.88) | 268(8.25) | 0.306 |
| Vital signs |  |  |  |  |  |  |
| HR_mean (times/min) | 79(69-90) | 87(74-100) | 81(70-93) | 79(69-90) | 77(68-87) | <0.0001 |
| SBP_mean(mmhg) | 113.93(104.2-125.67) | 105.94(97.32-118.07) | 112.67(103.32-122.67) | 115.96(106.67-129.54) | 115.08(105.68-126.5) | <0.0001 |
| DBP_mean (mmhg) | 59.5(52.57-66.6) | 56.16(50-63.23) | 56.31(50.15-63.57) | 56.5(50.32-63.23) | 61.38(54.73-68.28) | <0.0001 |
| MAP_mean (mmhg) | 76.01(69.55-83.58) | 72.27(66.33-79.62) | 73.24(67.65-81.24) | 75.12(69.05-82.96) | 77.72(71.27-84.72) | <0.0001 |
| RR_mean (times/min) | 18(16-21) | 20(17-23) | 19(17-22) | 19(17-21) | 18(16-20) | <0.0001 |
| TEMP_mean (℃) | 36.72(36.4-37.08) | 36.7(36.25-37.26) | 36.64(36.28-37.04) | 36.69(36.36-37.08) | 36.75(36.46-37.05) | 0.00014 |
| Spo2_mean (%) | 97.17(95.87-98.35) | 97.38(95.52-98.61) | 97.22(95.8-98.43) | 97.09(95.84-98.25) | 97.15(95.93-98.32) | 0.54987 |
| Laboratory parameters |  |  |  |  |  |  |
| AG_mean (mmhg) | 14(12-16) | 16(14-18.67) | 14.5(12.5-16.67) | 14(12-16) | 13.5(12-15) | <0.0001 |
| Bicarbonate_mean (mEq/L) | 24.5(22-27) | 22.25(19-26) | 25(22-28) | 25(22-27.5) | 25(22.5-27) | <0.0001 |
| Glucose_mean (mgl/dL) | 129(109-160.58) | 147(115.5-191) | 126(106.5-160) | 129(107-163.67) | 126.67(108.5-155) | <0.0001 |
| Sodium_mean (mmol/L) | 138.5(136-140.5) | 138(135-141.2) | 138.33(135.5-141) | 139(136.33-141) | 138.67(136.5-140.5) | 0.03219 |
| Potassium_mean (mmol/L) | 4.1(3.83-4.4) | 4.15(3.87-4.65) | 4.2(3.9-4.58) | 4.1(3.85-4.43) | 4.05(3.8-4.3) | <0.0001 |
| Calcium_mean (mgl/dL) | 8.5(8.1-8.9) | 8.25(7.79-8.75) | 8.45(8.05-8.85) | 8.53(8.2-8.9) | 8.53(8.15-8.9) | <0.0001 |
| Chloride_mean (mmol/L) | 104(101-107) | 103.5(99-107.67) | 103(99-107) | 104(100.5-107) | 104.5(102-107) | <0.0001 |
| Creatinine_mean (ng/dL) | 1.04(0.8-1.5) | 1.45(0.94-2.2) | 1.3(0.9-2.1) | 1.2(0.85-1.8) | 0.95(0.75-1.2) | <0.0001 |
| BUN_mean (mmol/L) | 21(14.5-33.88) | 33(21-50) | 30(20-46.67) | 26(17-40) | 17.5(13-25) | <0.0001 |
| WBC_mean (109 /L) | 10.3(7.9-13.55) | 13(9.5-17.85) | 10.15(7.3-13.35) | 9.77(7.47-12.8) | 10.1(7.9-12.9) | <0.0001 |
| Hemoglobin_mean (g/dL) | 11.4(10.1-12.8) | 10.85(9.8-12.25) | 10.45(9.5-11.7) | 10.9(9.85-12) | 11.8(10.45-13.2) | <0.0001 |
| Platelet_mean(109 /L) | 212(170-267) | 209(155-275) | 208(162.33-280.5) | 204.33(158.5-252.5) | 215(176-265.33) | 0.00027 |
| MCV_mean (fl) | 89.29±6.08 | 90.93±6.93 | 89.89±6.73 | 89.64±6.22 | 88.73±5.61 | <0.0001 |
| MCH_mean (pg) | 30.4(29.1-31.7) | 30.45(28.9-31.8) | 30.1(28.75-31.55) | 30.45(28.9-31.75) | 30.45(29.25-31.7) | 0.00664 |
| RDW_mean (%) | 14.1(13.3-15.27) | 14.9(13.8-16.6) | 15.1(14.05-16.77) | 14.5(13.65-15.8) | 13.75(13.1-14.6) | <0.0001 |
| Serum osmolarity_mean (mmol/l) | 297(292-304) | 304(295-313) | 301(293-309) | 300(294-307) | 296(292-301) | <0.0001 |
| Urine output | 1840(1129.5-2752.5) | 1120(575-1880) | 1591(923-2330) | 1835(1100-2687) | 2015(1335-3010) | <0.0001 |
| Medication use n (%) |  |  |  |  |  |  |
| Antiplatelet | 3804(70.97) | 414(59.40) | 463(67.49) | 516(70.78) | 2411(74.23) | <0.0001 |
| Anticoagulants | 4105(76.59) | 519(74.46) | 554(80.76) | 523(71.74) | 2509(77.25) | <0.0001 |
| Beta-blocks | 3697(68.97) | 342(49.07) | 480(69.97) | 509(69.82) | 2366(72.84) | <0.0001 |
| ACEI/ARB | 2742(51.16) | 143(20.52) | 343(50.00) | 390(53.50) | 1866(57.45) | <0.0001 |
| Statin | 3408(63.58) | 329(47.20) | 429(62.54) | 463(63.51) | 2187(67.33) | <0.0001 |
| Vasopressin | 213(3.97) | 110(15.78) | 27(3.94) | 16(2.19) | 60(1.85) | <0.0001 |

BMI body mass index; SOFA sequential organ failure score; COPD chronic obstructive pulmonary diseases; HR heart rate; SBP systolic blood pressure; DBP diastolic blood pressure; MAP mean arterial pressure; RR respiratory rate; TEMP temperature; SpO2 percutaneous oxygen saturation; AG anion gap; BUN blood urea nitrogen; WBC white blood cell; MCV mean corpuscular volume; MCH mean corpuscular hemoglobin; RDW red blood cell volume distribution width; ACEI angiotensin converting enzyme inhibitors; ARB Angiotensin Receptor Blocker.

**Supplementary Table S2** Details of feature ablation curves for XGBoost model

| Feature Number | **MCC** | **MCC_CI** |
| --- | --- | --- |
| 56 | 0.33783 | (0.31836421701989454, 0.357296607176745) |
| 55 | 0.335873 | (0.3169866845384318, 0.35476017122118897) |
| 54 | 0.333649 | (0.3165209605327568, 0.35077630146777317) |
| 53 | 0.333649 | (0.3165209605327568, 0.35077630146777317) |
| 52 | 0.331621 | (0.3126668518939744, 0.35057510764546) |
| 51 | 0.334073 | (0.3149229749510707, 0.3532229784980626) |
| 50 | 0.331425 | (0.3157083266341566, 0.3471420198916253) |
| 49 | 0.328258 | (0.3080528667151686, 0.3484628833438034) |
| 48 | 0.326162 | (0.30564477643584886, 0.34667976126279676) |
| 47 | 0.329206 | (0.31018983893354113, 0.3482221441301234) |
| 46 | 0.329247 | (0.3101827929977706, 0.3483104757323799) |
| 45 | 0.327682 | (0.3093486277165429, 0.34601602421861744) |
| 44 | 0.329618 | (0.3075752719225553, 0.35166010733678865) |
| 43 | 0.326285 | (0.30395616082721316, 0.3486131855210142) |
| 42 | 0.336092 | (0.3198758455965772, 0.35230869960405264) |
| 41 | 0.332549 | (0.3115943589881696, 0.3535035611551716) |
| 40 | 0.333154 | (0.31465362233980815, 0.35165343379635466) |
| 39 | 0.326775 | (0.31005686713137864, 0.34349300630571117) |
| 38 | 0.328242 | (0.3098790813651358, 0.34660391866181545) |
| 37 | 0.332497 | (0.31995059933237974, 0.3450429094900583) |
| 36 | 0.336493 | (0.31946035024320496, 0.3535255860494094) |
| 35 | 0.333583 | (0.3205687939553496, 0.34659815181158377) |
| 34 | 0.329666 | (0.31289380056560784, 0.34643887879333374) |
| 33 | 0.330327 | (0.31752887267563956, 0.3431243984930916) |
| 32 | 0.332033 | (0.32144051809315133, 0.3426247829634429) |
| 31 | 0.32856 | (0.31461978210765684, 0.3424992296193219) |
| **30** | **0.327391** | (0.3099908123339998, 0.3447913995043746) |
| 29 | 0.308149 | (0.2943185446528215, 0.32197953807072566) |
| 28 | 0.317645 | (0.3042909605112732, 0.33099888923363935) |
| 27 | 0.310567 | (0.2919956659470606, 0.3291386665211852) |
| 26 | 0.306635 | (0.2917646962538646, 0.32150527652562133) |
| 25 | 0.303206 | (0.27713055962169497, 0.32928054030726606) |
| 24 | 0.304881 | (0.28446017397581425, 0.3253021830156363) |
| 23 | 0.303243 | (0.28469712051747803, 0.3217894731704308) |
| 22 | 0.298543 | (0.28028015147570334, 0.3168050749296512) |
| 21 | 0.310695 | (0.287546063852732, 0.3338431475700427) |
| 20 | 0.293308 | (0.274754409833465, 0.3118609216984821) |
| 19 | 0.300392 | (0.28149202307112786, 0.31929157385412543) |
| 18 | 0.300037 | (0.2879320064548299, 0.31214298796191514) |
| 17 | 0.295239 | (0.2758959842522262, 0.3145826656394847) |
| 16 | 0.301882 | (0.282825058613137, 0.32093844399937144) |
| 15 | 0.282375 | (0.2633830661010322, 0.30136618157307055) |
| 14 | 0.276357 | (0.2579753251569237, 0.29473861134437884) |
| 13 | 0.264684 | (0.2476403573474477, 0.28172822880707155) |
| 12 | 0.266043 | (0.24322307149675143, 0.28886265439894737) |
| 11 | 0.258342 | (0.24172463049762796, 0.27495940766265653) |
| 10 | 0.256654 | (0.23505129960366306, 0.27825739255855914) |
| 9 | 0.255323 | (0.23460892327762503, 0.27603689536150866) |
| 8 | 0.263418 | (0.2368603355358142, 0.28997544597283265) |
| 7 | 0.231397 | (0.20440081207876143, 0.25839288614917466) |
| 6 | 0.202164 | (0.17881591467424884, 0.22551122133666435) |
| 5 | 0.188277 | (0.16477249580512987, 0.21178237163673014) |
| 4 | 0.167629 | (0.14780313694028768, 0.18745455269606692) |
| 3 | 0.157357 | (0.14231315446694853, 0.17240120376357942) |
| 2 | 0.150433 | (0.13901427056482796, 0.16185249441007477) |
| 1 | 0 | (NA, NA) |

MCC, Matthews correlation coefficient; CI, confidence interval.


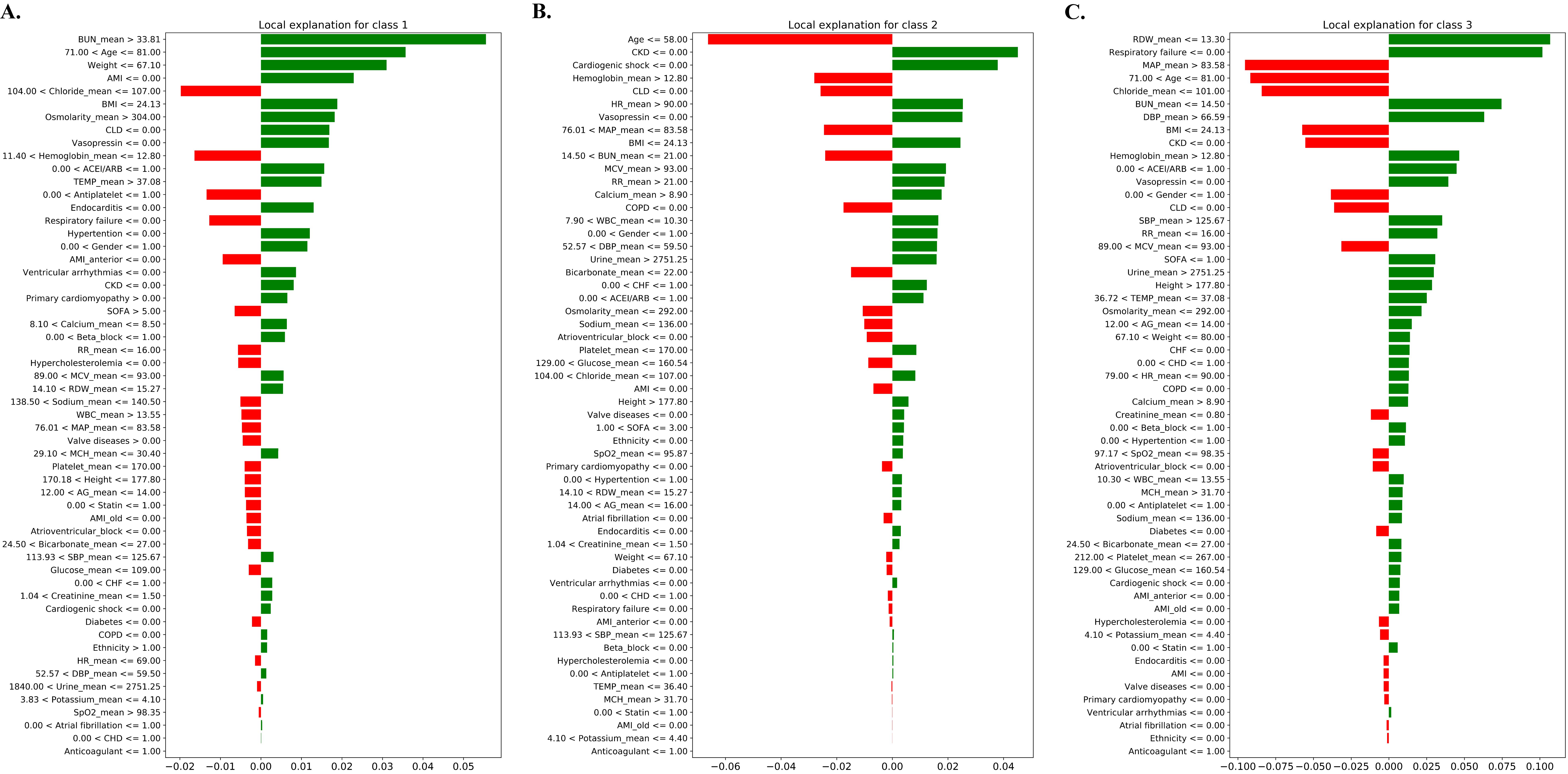


**Supplementary Figure S1**. The local interpretation of the XGBoost model. **(A)**. class 1 (30 days ~ 1 year); **(B)**. class 2 (1 year ~ 5 years); **(C)**. class 3 (≥5 years). The features of the green column make the model identify the sample as class x, while the features of the red column allow the model to identify the sample as non-class x. Since the sum of green column score exceeded red, the model finally identifies this sample as class x.
