# Supplementary figures and images for: Predicting the Prognosis of Patients in the Coronary Care Unit: A Novel Multi-Category Machine Learning Model Using XGBoost
Source: Front Cardiovasc Med. 2022 May 12;9:764629. doi: 10.3389/fcvm.2022.764629 (PMC9133425; doi:10.3389/fcvm.2022.764629)

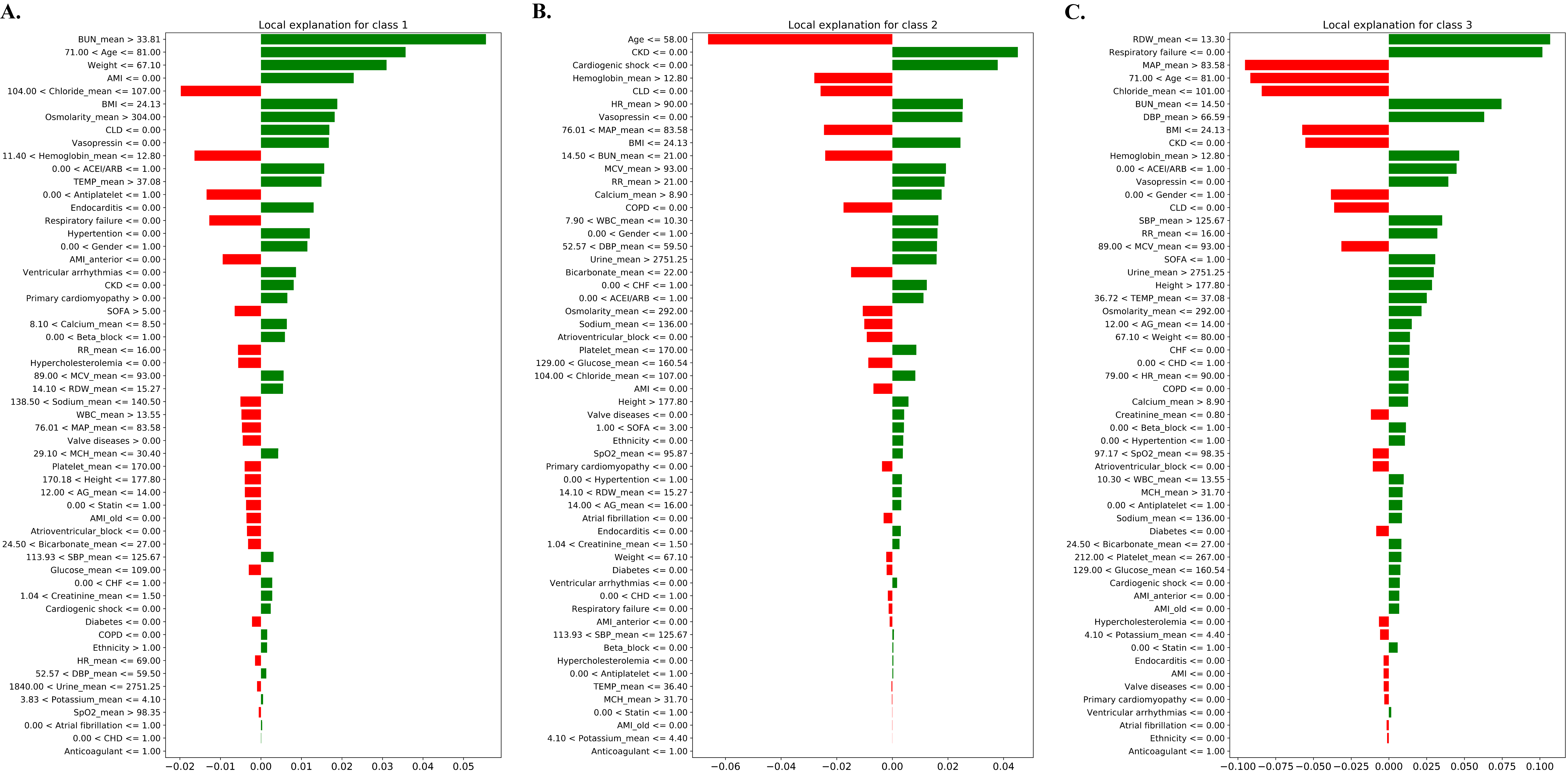

Supplement: Supplementary file 1 [file Data_Sheet_1.zip › Supplementary Figure S1.tif]
